# Modern Sociology

**Published:** 1898-11-05

**Authors:** 


### Modern Sociology.

By a Correspondent Within the Gates.

### SHORT PRISON SENTENCES.

A letter from the Secretary of the Howard Association to the editor of the *Times* of recent date, after dealing with juvenile criminals, proceeds to consider the question of short prison sentences. This is a subject which has to some extent occupied the attention of the Legislature in dealing with the provisions of the new Prisons Act, but they have considered it mainly in regard to the remission of part of the sentences on well-conducted prisoners, whereas the aspect of it, as affecting the aggregate of existing crime, is becoming one of very serious importance. There has been a marked tendency for a considerable time past, on the part especially of local magistrates, to render the sentences they are obliged to give as short as possible, and to substitute fines for imprisonment whenever the law allows them to do so. As Captain Anson says, "Sentences run lighter and lighter. I do not know what will be the end of it." The end of it up to the present time, by the testimony of his own most accurate statistics, admits of no question whatever. It has produced a disastrous increase in the crimes of burglary, housebreaking, and sacrilege—especially characteristic of professional criminals, which have increased with the shortening of the sentences to an alarming extent. The number of these particular crimes were—in 1876, 3,579; and in 1896 were more than double, viz., 7,308. These facts speak for themselves, and the secretary reaches the conclusion of the whole matter with the sensible remark that "it is no mercy to the professional criminal to give him a short sentence, while it is a crime against the honest and hardworking members of the community to liberate such an one amongst them." "It is no mercy to the criminal"—that statement will probably be disputed by our benevolent agitators for prison reform, and, therefore, we will bring a little practical experience from the internal life of those judicial establishments which will show it to be absolutely true. The two main objects in the incarceration of lawbreakers may be summarily given as consisting in the protection of society, and the benefit of the offender. Society can hardly be said to be protected from villains who go to prison for 7, 14, or 21 days, especially if they have the option of a fine. This is certain to be instantly paid for them by some obliging "pals," if they have not the wherewithal themselves; and thus, after a few hours detention, they are set free to resume with a light heart their predatory and other vicious propensities. The benefit to the criminal, of course, lies in the hope that his punishment and the good influences brought to bear upon him while it endures, may lead him to abandon the error of his ways, and become an honest and respectable member of the community. We are perfectly aware that most persons are entirely sceptical as to any good result on the character of an offender being rendered possible by the fact of his imprisonment. We can only say that, if space permitted, we could give numberless true and certain instances in which criminals have passed from the hands of those who laboured for their welfare within the prison walls, as totally changed men and women—who have realised not only the guilt but the mental and physical ruin involved in their evil courses, and who gladly avail themselves of the assistance given them and the plans laid out for them, whereby they may enter on a career of honest labour, and decent, cleanly living. The wholesome influences which have power to bring about such results are these: Attached to every prison there is a chaplain carefully chosen by the authorities for the qualities likely to make him most useful in that post; therefore usually a man of strong religious faith, of sound good sense, of warm sympathy without the slightest sentimentality, and possessing also the inestimable gift of tact, as strictly necessary in his vocation as in the case of the most intricate diplomacy; his whole soul, generally speaking, is in his work, and his life for the time being is entirely dedicated to it. He has free access to the prisoners; he can sit with them in their cells at suitable hours as much as he pleases. Only when he visits the women is there the restriction of a female officer's presence. He can make arrangements for them on their discharge, and keep in touch with them for years after, if he will. Nor is he the only, if perhaps the most potent, influence for good working on the prisoners throughout their detention. The governors of our gaols and convict establishments are, with scarcely an exception, men of high principle and earnest desire for the good of their fellow-creatures. Here, where their duty lies, they have unexceptionable opportunities of carrying out this object to the fullest extent. They generally make themselves thoroughly acquainted with the antecedents of their charges, and give them much wise and kindly advice as to their future career when they see signs of a disposition to reform. Nor does their beneficent influence rest there in the case of many amongst them. They often find means to offer the prisoners work on their discharge, or to recommend them in quarters where employment might be found Lastly, in addition to chaplains and governors, there are duly-appointed prison visitors, whose sole duty and object is to do all they can for the moral and social improvement of the criminals. At present these last assistants are still almost solely confined to the ladies appointed to visit the female prisoners, but we hope to see the day when they will be allowed to attend the male patients in the prison infirmaries also, and there seems to be no reason why volunteers of the opposite sex should not be allowed to work among the men, under the direction of the chaplain. All these agencies for good are, however, completely frustrated when the sentences are too short to admit of instruction being given or a salutary influence gained. Time passes quickly in the monotonous prison days, and a man who knows that he will be free again in a fortnight or a month does not trouble himself to listen to wise counsels when his mind is occupied with plans for resuming his old life in the near prospect of his discharge. It is far otherwise when long, dreary months are stretching out before him, with no distraction for his thoughts but such as he may find in intercourse with those inside the prison who desire to befriend him. In a word, we consider short sentences a mistaken policy and a false kindness.

